# Posterior Reversible Encephalopathy Syndrome Presenting with Atypical Findings: Report of Two Cases

**DOI:** 10.1155/2018/7835415

**Published:** 2018-11-25

**Authors:** Paolo Cerrone, Patrizia Sucapane, Rocco Totaro, Simona Sacco, Antonio Carolei, Carmine Marini

**Affiliations:** ^1^Department of Biotechnological and Applied Clinical Sciences, University of L'Aquila (DISCAB), Italy; ^2^Neurology Unit, San Salvatore Hospital, ASL 1, L'Aquila, Italy; ^3^Department of Life, Health and Environmental Sciences, University of L'Aquila (MeSVA), Italy

## Abstract

**Background:**

Posterior reversible encephalopathy syndrome (PRES) is characterized by a variable association of symptoms including headache, consciousness impairment, visual disturbances, seizures, and focal neurological signs. Treating the underlying cause usually leads to partial or complete resolution of symptoms within days or weeks. Brain MRI findings include hyperintensities on T2-weighted sequences and their reversibility on follow-up exams. We describe two patients, one with an atypical clinical presentation characterized by a severe and prolonged impairment of consciousness and the other with atypical neuroimaging findings.

**Case Presentation:**

The first patient was a 42-year-old woman, with a negative medical history, presenting with seizures, lethargy, and left hemiparesis, 60 hours after uncomplicated delivery. Brain MRI showed an atypical pattern of alterations, with patchy asymmetric distribution in all lobes. Symptoms completely resolved after twelve days. The second patient was a 59-year-old woman with a history of hypertension, presenting with severe impairment of consciousness, vision loss, and seizures. Symptoms partially resolved after three weeks.

**Conclusion:**

PRES is characterized by reversible symptoms and radiological findings. Brain MRI usually shows widespread oedema in white matter with typical patterns. The cases we described suggest that PRES may presents with atypical symptoms and radiological manifestations, mimicking other neurological conditions.

## 1. Background

Posterior reversible encephalopathy syndrome (PRES) is a clinicoradiological entity characterized by a variable association of symptoms including headache, consciousness impairment, visual disturbances, nausea/vomiting, seizures, and focal neurological signs [1].

PRES is associated with a series of conditions such as exposure to toxic agents, hypertension, infection/sepsis, preeclampsia/eclampsia, autoimmune diseases, and renal failure [1–3].

Symptoms may undergo partial or complete resolution within days or weeks if the underlying cause is treated, but in some cases the condition may progress to ischaemia, brain herniation, and death [4].

Neuroimaging findings include hyperintensities on Flair MRI sequences which may resolve at follow-up within days or several months [5, 6].

In this report, we present two patients, one with atypical neuroimaging findings and the other with atypical clinical presentation.

## 2. Case Presentation

### 2.1. Case 1

**Figure 1:**
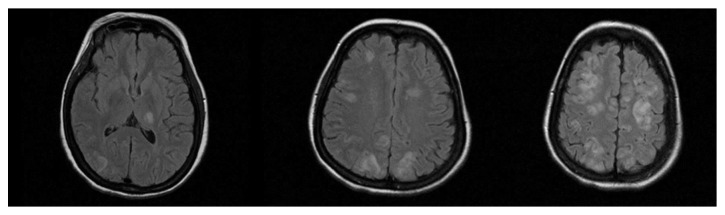
Case No. 1. Flair sequences showing multiple areas of hyperintensity in left thalamus and occipital and frontal lobes, extended to grey matter of the cortex.

**Figure 2:**
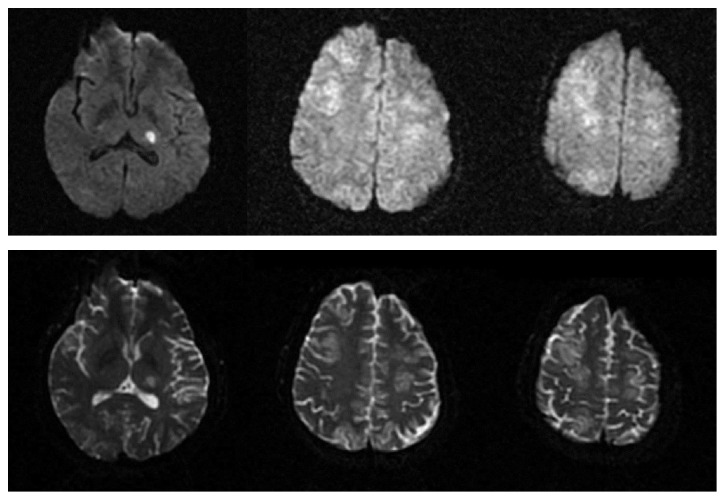
Case No. 1. DWI sequences (upper images) and ADC maps (lower images) showing diffusion restriction in left thalamus and mild signal hyperintensity in the same areas described in Figure 1.

A 42-year-old woman, in the 34th week of pregnancy, was admitted to the obstetrics unit of the university hospital with premature rupture of membranes. Her past medical history was negative. On admission, physical examination was unremarkable. Arterial blood pressure (ABP) was 110/70 mmHg, and temperature was normal. Complete blood cell count showed leukocytosis (12,430 cells/mmc) and severe microcytic hypochromic anaemia (Hb=6.9 mg/dL, Hct=25.2%, MCV=70.8 fL, and MCHC=27.4 g/dL). Anaemia was deemed chronic and was attributed to multiple uterine myomas. On admission, the patient was transfused with 4 units of blood. The following day, she underwent caesarian section. Therapy after surgery included hydration, low molecular weight heparin, antibiotics, nonsteroidal anti-inflammatory drugs (NSAIDs), ranitidine, calcium gluconate, cabergoline, and methylergometrine. After transfusion, Hb levels raised (Hb=9.3 mg/dL) and remained stable during the hospital stay. Arterial blood pressure values increased after blood transfusion (165/90 mmHg). On the third day after surgery, she presented a generalized tonic-clonic seizure which was treated with intravenous diazepam. Electroencephalogram registered after treatment showed rapid low amplitude waves without other relevant abnormalities. Neurologic examination revealed a lethargic status, with a mild right hemiparesis. Brain MRI showed multiple cortical and subcortical bilateral areas with hyperintense signal in T2, DWI, and Flair sequences, which did not show contrast-enhancement, involving not only posterior areas but also frontal lobes and right thalamus [Figures 1 and 2]. The last arterial blood pressure measurement was taken 4 hours before seizure (170/90 mmHg). The patient was transferred to the Neurology Department. The following day, the patient worsened, developing severe right hemiparesis. Blood pressure values were moderately high (160/100 mmHg). Transthoracic echocardiogram was normal. The patient was treated with antihypertensives (amlodipine, ramipril, amiloride, and hydrochlorothiazide), antiepileptics (levetiracetam 1000 mg bid), and osmotic therapy. Osmotic therapy was continued for 3 days. Three days after the reported seizure, the patient had improved and was alert and able to move right limbs against gravity. Brain CT showed widespread hypodensities in the same areas showing signal alterations in MRI imaging. Fundoscopy examination revealed an acute isolated retinal haemorrhage in left eye. Twelve days after the onset of symptoms, the patient had only a residual mild right hemiparesis. After 19 days, a control brain CT showed complete resolution of brain alterations.

### 2.2. Case 2

**Figure 3:**
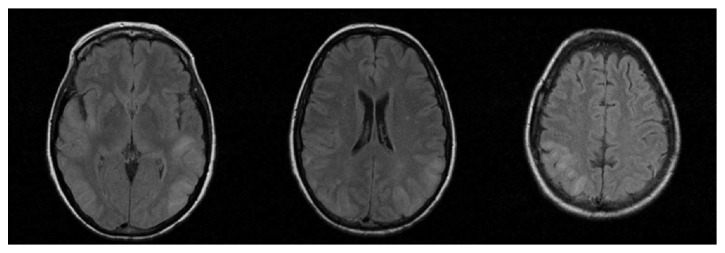
Case No. 2. Flair sequences showing alterations of signal in the white matter and cortex of the right temporal lobe and in both parietal and occipital lobes.

**Figure 4:**
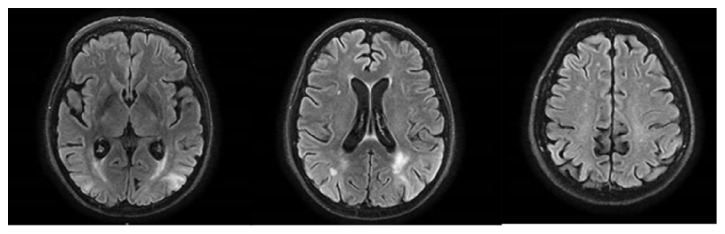
Case No. 2. One-month follow-up brain MR. Flair sequences showing alterations of signal in the white matter and cortex of the left temporal and occipital lobes and, to a lesser extent, of the right occipital lobe.

A 59-year-old woman, with a history of hypertension complicated with 2nd stage retinopathy and treated with bisoprolol, olmesartan, and amlodipine, was admitted to Emergency Department because of headache and transient loss of vision. Arterial blood pressure was 140/80 mmHg. Brain CT scan, visual field test, and neurological examination were normal. The patient improved quickly and was discharged to home the same day. After seven days, she presented with progressive confusion, loss of consciousness, and postural instability, with frequent falls. The patient was unable to maintain the standing position without support. She had also aimless movements, with repetitive, involuntary, purposeless, and slow movements of superior limbs. Neurologic examination revealed lethargy, impaired consciousness, loss of verbal comprehension, poor and inadequate verbal production, and dysphagia. Brain CT was normal. On admission, the patient had severe metabolic abnormalities including hypoglycaemia (49 mg/dL), hypokalemia (3.0 mEq/L), hypocalcemia (6.3 mg/dL), hypophosphoremia (1.3 mg/dL), hypomagnesemia (1.3 mg/dL), and hypoalbuminemia (3.38 g/dL). BP was 110/65 mmHg. Temperature was normal. Treatment was aimed at correcting metabolic imbalances and psychomotor agitation. The day after admission, the patient had two generalized tonic-clonic seizures. The last arterial blood pressure measurement was taken 6 hours before the first seizure (100/60 mmHg). After the crisis, arterial blood pressure was 140/90 mmHg. EEG showed widespread theta and delta subcontinuous activity. MRI showed hyperintensities in T2 and DWI sequences, localized in the white matter and cortex of the right temporal lobe and in both parietal and occipital lobes together with mild cerebral oedema. MR-Angiography did not show vascular abnormalities [Figure 3]. Brain CT performed after 48 hours showed hypodensity in the same brain areas. CSF examination was normal. The patient was treated with osmotics (mannitol 150 mL four times a day) and antiepileptics (levetiracetam 1000 mg bid). After 21 days from onset, verbal comprehension and consciousness considerably improved; the patient was alert and well oriented to space and people, though disoriented to time; she showed good verbal comprehension and answered appropriately to questions. She had motor slowing and difficulty in naming objects. One month after admission, brain MRI showed a partial regression of signal alterations in T2 and Flair sequences, with persisting hyperintensities only in left temporal and occipital lobes and, to a lesser extent, in the right occipital lobe [Figure 4].

## 3. Discussion

These case reports described two patients with reversible clinical and radiological manifestations that were attributed to PRES. Other causes, as infectious diseases, inflammatory processes, and toxic exposures, were excluded.

In the first patient, the aetiology of PRES could be linked to late postpartum eclampsia (LPE), as described in other studies [5, 7]. In LPE, the onset of convulsions occurs more than 48 hours, but less than 4 weeks, after delivery. LPE frequently occurs in women without signs of a preeclamptic syndrome, including proteinuria. The delayed onset and the atypical presentation may lead to misdiagnosis in LPE [7, 8]. The patient had no history of hypertension but arterial blood pressure values and fundoscopy findings suggested the occurrence of an acute hypertensive episode. Fundoscopy examination revealed an acute isolated retinal haemorrhage in left eye. Ophthalmologist interpreted this finding as suggestive of an acute hypertensive episode.

Imaging alterations are typically bilateral and localized in the posterior white matter, but frontal and temporal lobes are often involved. Atypical localizations are cortical grey matter, basal ganglia, brainstem, and cerebellum [6]. In the first case, there was an atypical neuroimaging pattern, with vasogenic oedema involving white matter and cortex of all lobes and right thalamus with patchy distribution. Widespread involvement of the frontal cortex may explain the stroke-like clinical syndrome. Distribution of lesions in basal ganglia was described in patients with PRES due to eclampsia, but only rarely [9].

Unlike what the name of the syndrome suggests, predominantly posterior distribution of the lesions affects only 22% of patients with PRES. Frontal and temporal lobes are often involved. Lesions were asymmetric in 28% of patients [3].

In the second patient, PRES was probably linked to hypertension. The patient had a history of complicated hypertension and three admissions to the Emergency department for hypertensive crisis. Seven days before main clinical syndrome due to PRES, the patient experienced a short-duration episode characterized by headache, transient bilateral loss of vision, and hypertensive crisis. This is an unusual presentation of PRES and it could have led physicians to consider other diagnoses. This transient PRES-like syndrome might be the clinical manifestation of transient vascular dysregulation in the vertebrobasilar territory, without vasogenic oedema. When the main episode developed after seven days, other diagnoses, including stroke, cerebral venous thrombosis, and encephalitis, were considered. However, a cerebrospinal fluid analysis did not show abnormalities, the patient had no signs of infective diseases and Brain MRA did not show any abnormalities. There was a marked improvement of symptoms and a partial reduction of typical MR abnormalities on 1-month follow-up. During hospitalization, arterial blood pressure values were in the normal range, but there may have been episodes of acute hypertension between the first episode and the onset of PRES. The patient had also severe hypomagnesaemia that has been associated with PRES [10]. Clinical presentation was atypical with severe and prolonged impairment of consciousness without clinical improvement within the first weeks. On brain MRI, there was a typical dominant parietal-occipital pattern, with alterations suggestive of vasogenic oedema. The classical pathophysiological mechanism underlying the posterior dominance pattern is linked to the scarce sympathetic innervation in the vascular territory of basilar artery and its branches. Normally, sympathetic activation leads to arteriolar constriction in responding to severe hypertension. A relative lack of sympathetic innervation may lead to an increased risk of cerebral vasogenic oedema during hypertensive crisis [2]. This may explain the distribution of the lesions in the second case, but it does not fit in the first case. In the first case, lesions were patchy, asymmetric, and distributed in basal ganglia and occipital and frontal lobes, both in white and grey matter. Different arteries and branches supply these areas, so the hypertension with failed autoregulation hypothesis does not explain the case. Other possibilities, as reported by Bartynski et al., may explain the pathophysiological process. Proinflammatory cytokine response leading to endothelial activation may be the cause of cerebrovascular dysregulation in eclamptic women [11].

Treatment of PRES includes symptomatic management and early correction of underlying cause of PRES [1]. In the cases described, the first measures taken were aimed at controlling arterial blood pressure and restoring fluid and electrolyte balance. Symptomatic treatment includes control and prevention of seizures. The first suspected diagnosis based on symptoms and acute phase brain CT was ischaemic stroke and we took the decision to treat both patients with short-duration cycles of osmotics due to alterations of consciousness and to the presence of signs of cerebral oedema on neuroimaging. Even if there is no strong evidence for the use of osmotic therapy in PRES, this syndrome is characterized by cerebral blood perfusion abnormalities resulting in vasogenic cerebral oedema [1] and osmotic therapy might play a role in the resolution of oedema.

In conclusion, the cases we presented suggest that PRES may present with atypical imaging and clinical presentation and that high blood pressure episodes may be overlooked. PRES may mimic other neurological conditions. An important criterion to identify PRES is the reversibility of symptoms and radiological findings, but this may occur later than expected. Collecting carefully the medical history and searching possible signs of the associated conditions in suspected PRES may help in the diagnostic process, avoiding unnecessary and potentially dangerous treatments.
